# Ascaris worm in the intercostal drainage bag: inadvertent intercostal tube insertion into jejunum: a case report

**DOI:** 10.1186/1749-8090-5-125

**Published:** 2010-12-08

**Authors:** Prashant N Mohite, Jitendra H Mistry, Harshad Mehta, BS Patra

**Affiliations:** 1 Department of Cardiothoracic & Vascular Surgery, SSG Hospital & Medical College, Sayajiganj, Vadodara, Gujarat, India, 390001

## Abstract

Inadvertent insertion of the intercostal tube into abdomen is not rare. It can present by different ways. In the present case an Ascaris worm crept into the intercostal drainage bag to reveal the false passage of the tube.

## Case report

**Figure 1:**
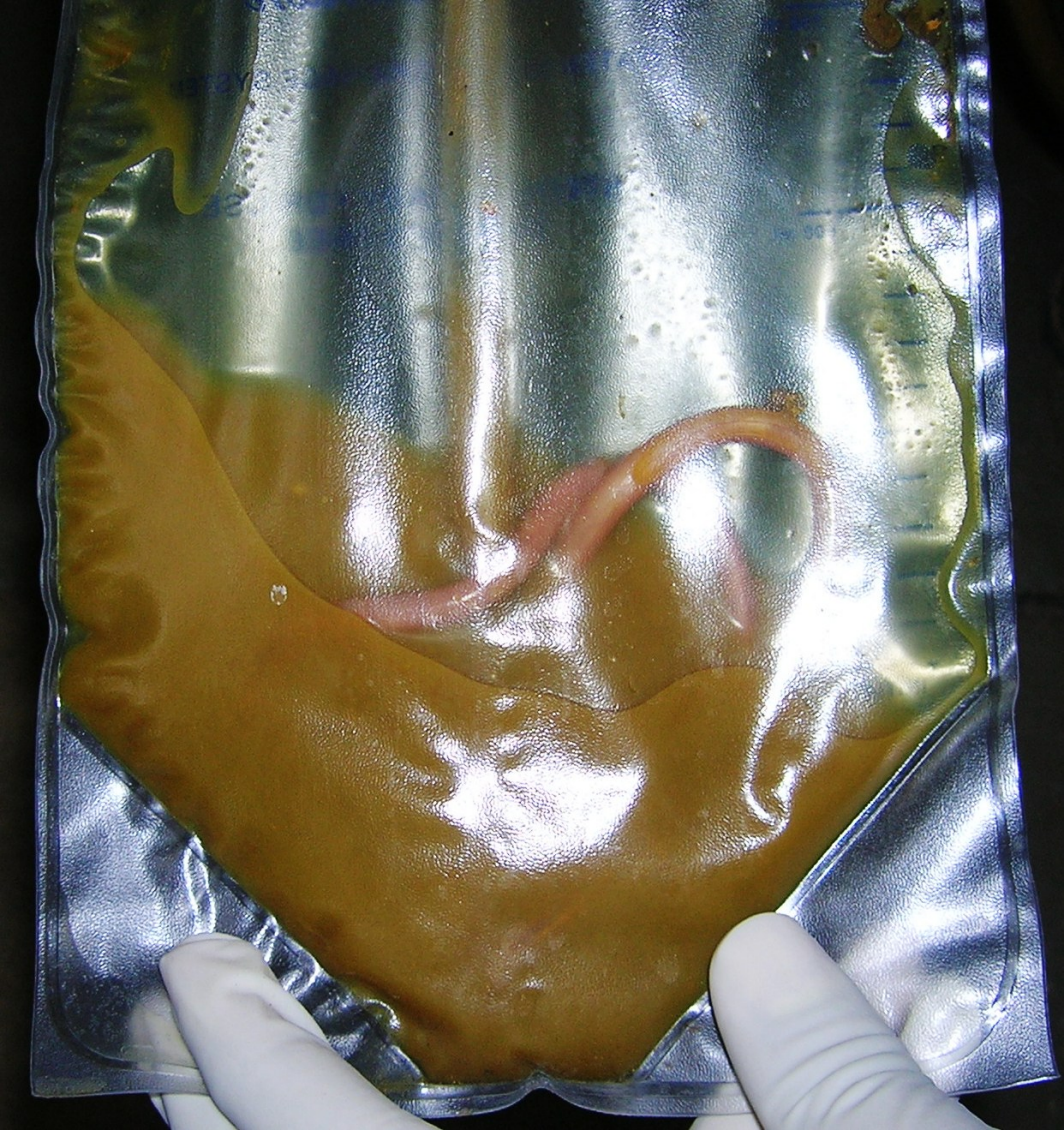
**An Ascaris worm in the intercostal drainage bag**.

A middle age man presented in the emergency department late night with the history of recent blunt trauma over left chest complaining of breathlessness and chest pain. Air entry was absent on the left side of chest and x-ray chest showed left pneumothorax with collapsed lung. Emergency intercostal tube drainage was planned. One and half centimeter skin was incised at fifth intercostal space in anterior axillary line. An artery forceps was inserted through the incision making its way through intercostal muscles till parietal pleura gave way. The forceps was removed and the index finger was inserted into the wound to confirm its entry into pleural cavity. The 32 French intercostal tube was held into the artery forceps and thrust through the incision into the left pleural cavity. Approximately half liter of blood was drained through the tube. Tube was fixed after confirming the air fluid column movement in the tube. Another half liter of dark blood was drained overnight. Next morning, chest x-ray showed the tube in the left chest directing downward into the costophrenic angle above the diaphragm. The left lung was well expanded and there was no air under diaphragm. In the afternoon, an Ascaris worm was noticed in the intercostals drainage bag along with fifty milliliters of blood mixed with bile (See Figure 1). The patient had no abdominal complaints, no air was noticed under diaphragm on erect abdominal x-ray and there was no free fluid in peritoneal cavity on ultrasonography of abdomen. Emergency exploratory laparotomy was planned suspecting bowel injury following breach of diaphragm by intercostal tube. In the laparotomy, intercostal tube was found perforating the left dome of diaphragm with tip entering into the loop of jejunum. The tube was repositioned inside the left chest and diaphragmatic rent was repaired with 2-0 polypropelene. Jejunal perforation was closed in two layers using Polyglactin (Vicryl) suture. Chest tube was removed on second day of operation and the patient made swift recovery.

## Discussion

Pneumothorax is present in about one fifth of the blunt chest trauma cases. Insertion of an intercostal tube drainage is one effective treatment and significant morbidity can be avoided by prompt pleural decompression using proper techniques [1]. Both ventral and lateral approaches are equally preferred by the clinicians and no statistically significant difference between the two approaches for functional malposition is observed [2]. Inadvertent abdominal insertion of the intercostal tube is not rare but it is diagnosed immediately by absent air column movement in tube as well as with development of pneumoperitoneum and abdominal symptoms. Injury to the stomach or bowel may bring ingested or digested food particles into the chest tube [3]. In present case, the inadvertent entry of chest tube into jejunal loop was concealed, may be, because of snug fitting of tube into jejunum which prevented leak of intestinal air and fluid into peritoneum. The air column movement was present in the tube as the proximal holes in the tube were in left chest. The drainage of bile was not apparent initially as it was mixed with more quantity of blood in chest. It was revealed only when an Ascaris worm made its way out through the tube.

## Conclusion

Close observation of the chest tube drainage bag contents should be the routine practice.

## Consent

Written informed consent was obtained from the patient for publication of this case report and accompanying images. A copy of the written consent is available for review by the Editor-in-Chief of this journal.

## Competing interests

The authors declare that they have no competing interests.

## Authors' contributions

PNM: Manuscript preparation, design; JHM: Manuscript review; HM: Concept; BSP: Literature search. The manuscript has been read and approved by all the authors and the requirements for authorship have been met, and each author believes that the manuscript represents honest work.
